# Application of Zearalenone (ZEN)-Detoxifying *Bacillus* in Animal Feed Decontamination through Fermentation

**DOI:** 10.3390/toxins11060330

**Published:** 2019-06-08

**Authors:** Shiau-Wei Chen, Han-Tsung Wang, Wei-Yuan Shih, Yan-An Ciou, Yu-Yi Chang, Laurensia Ananda, Shu-Yin Wang, Jih-Tay Hsu

**Affiliations:** 1 Department of Animal Science and Technology, National Taiwan University, No. 50, Lane 155, Sec 3, Keelung Rd, Taipei 10673, Taiwan; d98626002@ntu.edu.tw (S.-W.C.); rumen0808@gmail.com (H.-T.W.); g2299015@gmail.com (Y.-A.C.); 2 Animal Resource Center, National Taiwan University, No. 118, Lane 155, Sec 3, Keelung Rd, Taipei 10673, Taiwan; weiyuanshih@ntu.edu.tw; 3 Graduate Institute of Biotechnology, Chinese Culture University, No. 55, Hwa-Kang Rd, Taipei 11114, Taiwan; yychang@ntu.edu.tw (Y.-Y.C.); lq_lin@ymail.com (L.A.); sywang@faculty.pccu.edu.tw (S.-Y.W.)

**Keywords:** zearalenone, biological detoxification, *Bacillus*, fermentation

## Abstract

Zearalenone (ZEN) is an estrogenic mycotoxin which can cause loss in animal production. The aim of this study was to screen *Bacillus* strains for their ZEN detoxification capability and use a fermentation process to validate their potential application in the feed industry. In the high-level ZEN-contaminated maize (5 mg·kg^−1^) fermentation test, B2 strain exhibited the highest detoxification rate, removing 56% of the ZEN. However, B2 strain was not the strain with the highest ZEN detoxification in the culturing media. When B2 grew in TSB medium with ZEN, it had higher bacterial numbers, lactic acid, acetic acid, total volatile fatty acids, and ammonia nitrogen. The ZEN-contaminated maize fermented by B2 strain had better fermentation characteristics (lactic acid > 110 mmol·L^−1^; acetic acid < 20 mmol·L^−1^; pH < 4.5) than ZEN-free maize. Furthermore, B2 also had detoxification capabilities toward aflatoxins B1, deoxynivalenol, fumonisin B1, and T2 toxin. Our study demonstrated differences in screening outcome between bacterial culturing conditions and the maize fermentation process. This is important for the feed industry to consider when choosing a proper method to screen candidate isolates for the pretreatment of ZEN-contaminated maize. It appears that using the fermentation process to address the ZEN-contaminated maize problem in animal feed is a reliable choice.

## 1. Introduction

Zearalenone (ZEN) is a nonsteroidal estrogenic mycotoxin produced by *Fusarium* species on cereal crops grown in warm, humid climates. Its contamination mostly occurs in preharvest periods rather than in storage periods [1]. Maize is susceptible to *Fusarium* infection and contamination by ZEN [2,3]. ZEN is classified as an endocrine disruptor, due to its estrogenic activity, which can disrupt the estrous cycles in animals [4]. Swine are more sensitive to ZEN effects than other species [2,5]. ZEN in diet as low as 1 ppm may lead to hyperestrogenic syndrome in gilts [5]. In addition, ZEN and its metabolites may affect humans through egg, milk, or meat. The potential threats of ZEN should not be ignored. 

The mycotoxin’s structure plays a key role in its toxicity [2,6]. When the lactone ring of ZEN is cleaved by esterase or lactonohydrolase, its ability to bind to estrogen receptors is compromised [7]. Various strategies have been developed to detoxify mycotoxins, but most of them have shown disadvantages in animal feed applications [6,8]. Several studies have suggested that biological detoxification is effective, specific, and safe in the decontamination of animal feed [1,6,9]. Biological detoxification depends on microorganism enzyme action to degrade mycotoxins and microbial cell walls to adsorb mycotoxins. In recent years, the mycotoxin detoxification ability of *Bacillus* spp. has been noticed. *Bacillus* spp. is Gram-positive, spore-forming bacteria. Many *Bacillus* spp. have been proposed for their qualified presumption of safety (QPS) status by the EFSA such as *B. amyloliquefaciens, B. licheniformis*, and *B. subtilis*. They are often utilized to produce enzymes or used as microbial feed additives [10]. With all these characteristics, *Bacillus* spp. are one of the safe and perfect candidates to act as a ZEN detoxification agent in animal feed.

Fermented liquid feed (FLF) is widely used in the animal industry and has many advantages. It can be produced by fermentation of the complete feed or by fermentation of cereals before being incorporated with other feed ingredients [11]. FLF has been shown to improve the growth performance of pigs and to decrease the incidence of enteric diseases in pigs by lowering enteric pathogen numbers [11,12]. The objective of this study was to screen ZEN-detoxifying *Bacillus* (ZDB) strains and put the candidate strains through fermentation of ZEN-contaminated maize to evaluate their field application potential for animal feed.

## 2. Results

### 2.1. Screening of Bacillus Strains for ZEN Detoxification Potential

In this study, 106 isolates were successfully isolated from fermented soybean products, soil, sewage, rumen fluid, and ruminant feces. All isolates were included in the ZEN detoxification capability test. The results showed that 14 isolates had a markedly greater ZEN detoxification ability than the others (Table 1). Out of these 14 isolates, seven isolates were from fermented soybean products, one from sewage, and the remaining from soil. The 16S rRNA gene sequence analysis indicated that these 14 isolates shared the highest identities with *Bacillus* spp. In the enterotoxin detection assays, nine out of the 14 strains were identified as enterotoxin producers. The five non-enterotoxin-producing ZEN-detoxifying *Bacillus* (ZDB) strains were kept for further study (Table 1).

### 2.2. ZEN Detoxification Capability, Adsorption Ability, and Degradation Ability of ZDB Strains

The detoxification rate of strain B4 was 58%, followed by strain 17,441 (52%) in the culturing condition (Table 2). The detoxification rate of the other ZDB strains were between 28% and 43%. The adsorption ability of strain 17,441 (47%) was significantly higher than all of the other ZDB strains (Table 2) (*p* < 0.05). No significant differences were found between the degradation rate of all ZDB strains and type strain 17,441. The degradation rate of each ZDB strain (B1–B5) and strain 17,441 were 24, 35, 27, 31, 33, and 31%, respectively.

### 2.3. Enzymatic Profile of ZDB Strains

The API ZYM assay showed that most enzyme activities were positive (Table 3). All strains demonstrated esterase activity, which is linked to ZEN degradation ability. The B1 and B2 strains showed stronger esterase activity than the other strains, which was consistent with the high level of ZEN degradation observed with the B2 strain (Table 2).

### 2.4. Detoxification of ZEN-Contaminated Maize by ZDB Strains

To ensure that the unsuccessful detoxification in ZEN-contaminated maize was not due to fermentation failure, the bacterial growth after 24 h and 48 h of fermentation were checked. At the beginning, the total number of bacteria was approximately 10^5^ to 10^6^ cfu·mL^−1^. After 24 h, the bacterial number increased to 10^9^ cfu·mL^−1^, except for strain B1 (Table 4). After 48 h, the bacterial number of most strains slightly decreased and remained at 10^8^ to 10^9^ cfu·mL^−1^ (Table 4). All of the ZDB strains significantly reduced the ZEN content in maize after fermentation (Table 4). The B2 strain had the highest detoxification rate, removing 56% of the ZEN from the maize (*p* < 0.05). Conversely, strain 17,441 had the lowest detoxification rate. Among all ZDB strains, B2 strain showed the best detoxification capability, and it was selected for further research. The *gyrB* gene sequencing identified the B2 strain as *B. subtilis*.

### 2.5. The Effect of ZEN on B2 Strain Growth in TSB Medium

The B2 strain was inoculated in TSB with or without ZEN to explore the influence of ZEN on its growth and metabolism. All parameters were increased significantly when B2 strain was inoculated in TSB with ZEN, including bacterial number, pH, NH_3_-N, lactic acid, acetic acid, and total VFAs (Table 5). The results indicated that the growth rate of B2 strain was stimulated by ZEN, and the production of metabolites was enhanced.

### 2.6. The Effect of ZEN on Fermentation Characteristics of B2 Strain in Maize

There was no statistically significant difference in the bacterial number of B2 strain between ZEN-free and ZEN-contaminated maize during 48 h and 72 h of fermentation (Table 6). After 48 h, the lactic acid concentration of fermented product from ZEN-contaminated maize was significantly higher than ZEN-free maize, resulting in a lower pH (Table 6) (*p* < 0.05). After 72 h, fermented ZEN-free maize product had significantly less NH_3_-N and more acetic acid (Table 6). Overall, the fermented product of ZEN-contaminated maize had better fermentation characteristics (lactic acid > 110 mmol·L^−1^; acetic acid < 20 mmol·L^−1^; pH < 4.5) after 72 h of fermentation than ZEN-free maize.

### 2.7. Other Mycotoxins’ (AFB1, DON, FB1, and T2 Toxin) Detoxification

**Figure 1:**
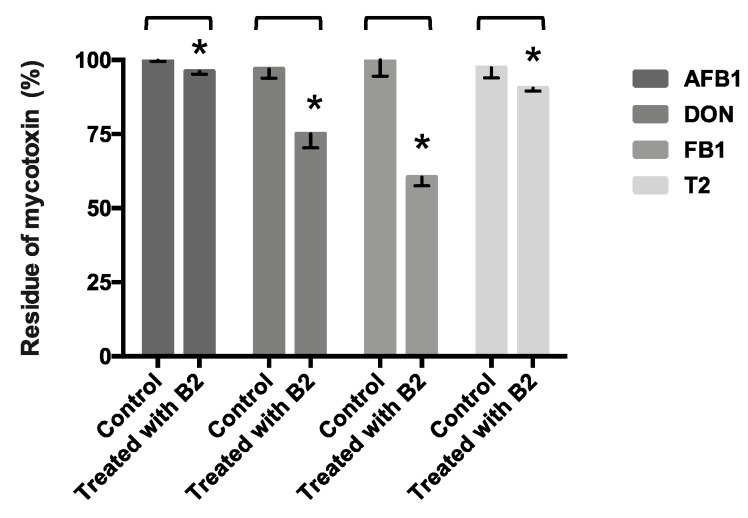
The mycotoxins detoxification of B2 strain in culturing condition. The values represent the mean ± SD of the quadruplicate experiments. Asterisks indicate that the residual percentage is significantly different among treatments (*p* < 0.05).

Figure 1 illustrates that B2 strain significantly reduced the AFB1, DON, FB1, and T2 toxin content in TSB medium after 24 h cultivation (*p* < 0.05). The detoxification rates of AFB1, DON, FB1, and T2 toxin were 3.8, 25.0, 39.5, and 9.5%, respectively. The results showed that B2 strain had detoxification capability toward multiple mycotoxins.

## 3. Discussion

The extreme weather conditions caused by global warming increase the risk of mycotoxin contamination of cereal crops. In some regions, the impact of ZEN on agriculture is the second highest impacting factor after aflatoxins [13]. It is virtually impossible to avoid the production of mycotoxins. Therefore, it is necessary to develop an effective detoxification strategy for mycotoxin-contaminated food or feed [3]. During the past decades, some microorganisms and their enzymes have been verified to detoxify ZEN, including fungi, yeast, and bacteria [14,15,16,17,18,19,20,21,22,23]. However, most of these microorganisms are not allowed for application in food and feedstuff. There are still some doubts surrounding the toxicity of microbial detoxification products and undesirable side effects from fermentation involving non-native microorganisms [24]. Based on this reason, the present study aimed to screen *Bacillus* spp. which are commonly applied in animal feed. 

*Bacillus* spp. can form endospores which can resist unfavorable environment conditions such as heat, chemicals, and radiation [25]. In this study, all samples were cultured for 72 h in TSB with polymyxin B and isolated after heat treatment. Because of the endospore formation, *Bacillus* spp. should survive after these treatments. Based on 16S rRNA gene sequence analysis, 14 isolates with better ZEN detoxification ability were identified as *Bacillus* spp. (Table 1). 

Generally, there are two mechanisms through which microbes detoxify ZEN: degradation and adsorption. In the present study, all of the tested ZDB strains demonstrated esterase activity (Table 3), which is linked to their ZEN degradation ability. The B2 strain was one of stronger detoxifying strains (Table 2), and its detoxification rate was the highest out of all of the strains when tested with the ZEN-contaminated maize (Table 4). Previous research indicated that the efficiency of the ZEN degradation ability of *Bacillus* also depends on the initial ZEN concentration [26]. When the initial ZEN concentration was 0.02 and 5 mg L^−1^, the degradation rate was found to be 100 and 18%, respectively [27]. In the present study, the initial ZEN concentration was 5 mg·L^−1^ for all experiments. The degradation rates of the ZDB strains were between 24 and 35 % which is greater than previously reported (Table 2). According to Reddy et al., the amounts of ZEN in grain ranges from a few μg·kg^−1^ to thousands of μg kg^−1^ worldwide [13]. The ZDB strains of the present study performed well at higher level ZEN contamination (mg·kg^−1^). 

All of the ZDB strains tested in this study were able to adsorb ZEN (Table 2). Previous research has suggested that the adsorption mechanism of *Bacillus* is similar to that of *Lactobacillus* because both are Gram-positive bacteria and have the same cell wall characteristics [27]. ZEN is mainly adsorbed by the surface hydrophobicity and the carbohydrate components of the *Lactobacillus* cell wall [28]. Therefore, ZEN adsorption by ZDB strains may rely on the same components. The high concentration of ZEN promotes the bacterial cell wall to contact with ZEN, which increases ZEN adsorption [29]. Although high concentration of ZEN was used in ZEN adsorption test, the ZEN adsorption rate of ZDB strains were still low. The data suggested that poor adsorption capacity of the ZDB strains may favor contact of ZEN with enzymes. In fact, adsorption of ZEN by microorganisms does not really remove the ZEN, which may be released back into the digestive tract when the digestive fluid continues to flush the bacterial surface [30]. It is worth noting that the ZEN detoxification capability of strain 17,441 in TSB was the second highest (52%) in the culturing condition (Table 2). Strain 17,441 also demonstrated the greatest adsorption capability (Table 2). However, strain 17,441 exhibited a lower detoxification rate than the other ZDB strains in the ZEN-contaminated maize detoxification experiment (Table 4). Therefore, the detoxification capability of a given strain in culturing condition is not necessarily the same as in the feedstuff fermentation process. 

Suitable microorganisms must be easily applied in feed, and their detoxifying action must be fast enough in complex environments, e.g., the gastrointestinal tract or feed pretreatment. The pH has a significant impact on ZEN degradation [26], and the gastrointestinal tract environment may not be suitable for certain microorganisms and their enzymatic reactions. Moreover, previous research has found that acid-treated *Bacillus* adsorbed less ZEN than untreated cells [27], implying that low pH sites, such as the gastrointestinal tract, are unfavorable for the microbial degradation and adsorption of ZEN. ZEN is absorbed by intestinal epithelium within 30 min after entering the duodenum [31]. Because intestinal absorption is quick, ZEN detoxification must take place rapidly or be accomplished before feeding [6]. Therefore, application of the microbial fermentation process for animal feed detoxification could be a suitable strategy. 

The fermentation process can cause loss of nutrients. Some studies suggest that FLF should be produced by fermenting the cereal ingredient instead of the complete feed [11,12]. Furthermore, fermentation of cereals often leads to a more rapid fermentation than compound feed. In the present study, maize contaminated with high level of ZEN (5 mg·kg^−1^) was used as a substrate, and ZDB strains grew normally and retained their detoxification capacity (Table 4). 

In order to control the growth of pathogenic bacteria, FLF should have a pH below 4.5 [32,33]. FLF should contain at least 75 mmol·L^−1^ lactic acid to avoid the growth of *Salmonella* spp. [33] and above 100 mmol·L^−1^ to decrease the number of enterobacteria [11]. FLF also has beneficial effect on daily gain, feed intake, and feed efficiency. However, a high concentration of acetic acid would make the FLF less palatable [33]. The acetic acid concentration of FLF should less than 40 mmol·L^−1^ [33]. In the present study, both the fermentation products of ZEN-free maize and ZEN-contaminated maize had good fermentation characteristics (lactic acid > 110 mmol·L^−1^; acetic acid < 20 mmol·L^−1^; pH < 4.5) after 72 h of fermentation. The results indicated that the B2 strain may be a suitable candidate for ZEN detoxification by fermentation. 

Bacteria have developed complicated regulation systems to obtain nutrients from a wide range of sources. In *B. subtilis*, catabolite control protein A (ccpA) and codY are the major global regulators of transcription connected with carbon metabolism involving in the synthesis of lactic acid and acetic acid [34,35]. In the present study, the presence of ZEN significantly increased the lactic acid concentration in B2 strain (Table 5 and Table 6). It is speculated that ZEN may have affected ccpA and codY of B2 strain, which needs further investigation. A significant increase in NH_3_-N concentration (Table 5 and Table 6) may imply that ZEN also affects protein metabolism in B2 strain. When B2 strain was inoculated in TSB with ZEN, the bacterial number was increased significantly (Table 5). It is worth noting that ZEN can be a potential growth promoter for B2 strain.

From a practical perspective, animal feed may not be only contaminated by one kind of mycotoxin [3]. The additive or synergistic interactions of co-occurring mycotoxins might lead to unpredictable toxicity [36]. An appropriate detoxification strategy should be able to detoxify multiple mycotoxins. The B2 strain has been confirmed to have the ability to detoxify AFB1, DON, FB1, and T2, which is very appealing.

## 4. Conclusions

Overall, the B2 strain may be a suitable candidate for ZEN detoxification by fermentation before feeding because it demonstrated strong esterase activity and exhibited the highest detoxification capability in maize with a high level of ZEN (5 mg·kg^−1^ maize).

## 5. Materials and Methods 

### 5.1. Chemicals

HPLC grade methanol and acetonitrile were purchased from Sigma-Aldrich (St. Louis, MO, USA), chloroform and polymyxin B from Merck (Darmstadt, Germany), ZEN from Enzo Biochem (Farmingdale, NY, USA), aflatoxin B1 (AFB1), deoxynivalenol (DON), fumonisin B1 (FB1) and T2 toxin from Sigma-Aldrich (St. Louis, MO, USA), and tryptic soy broth (TSB) and tryptic soy agar (TSA) from Acumedia (Lansing, MI, USA). 

### 5.2. Isolation of Bacillus Strains

In this study, fermented soybean products, soil, sewage, rumen fluid, and ruminant feces were collected for the isolation of ZEN-detoxification *Bacillus* (ZDB). One gram of each sample was suspended in 10 mL TSB containing polymyxin B (100,000 IU L^−1^) and incubated for 72 h at 37 °C. All of the cultured samples were heated in a water bath for 15 min at 80 °C and then spread on TSA with polymyxin B and incubated at 37 °C for 24 h. Individual colonies from each plate were collected for further screening.

### 5.3. Screening of ZEN Detoxification Potential Strains

All isolates were inoculated at 1% (*v/v*) in TSB containing 5 mg L^−1^ ZEN for 24 h at 37 °C. Then, samples were centrifuged for 20 min at 8000× *g* at 4 °C; the supernatants were collected and extracted with an equal volume of chloroform and sonicated for 30 min. The organic phase was separated by centrifugation (500× *g* for 10 min at 25 °C) and dried with nitrogen gas at 63.5 °C. The residues were re-dissolved in 1 mL methanol and concentrated to 1/5 of the original volume by centrifugal vacuum concentrator (5301 VacuFuge, Eppendorf®, Hamburg, Germany) at 60 °C, then filtered through a 0.22 μm nylon syringe filter before loaded into HPLC (LC-2000Plus, JASCO, Tokyo, Japan) with a fluorescence detector (excitation and emission wavelengths were 274 and 440 nm) and the Luna® 5 μm C18(2) 100-Å, LC column (250 × 4.6 mm) (Phenomenex, Torrance, CA, USA) to detect residual ZEN. The mobile phase was acetonitrile solution (50:50, *v/v*).

### 5.4. Bacterial Strain Identification

The *Bacillus* isolates were identified through 16S rRNA gene sequencing. DNA was extracted from each isolate using the DNeasy plant mini kit (Qiagen, Valencia, CA, USA). The PCR products were sequenced using the BigDye terminator v3.1 cycle sequencing kit (Applied Biosystems, Foster City, CA, USA), and sequencing was performed on a DNA Analyzer (3730XL, Applied Biosystems, Foster City, CA, USA). The sequences (approximately 1500 bp) were compared with 16S rRNA gene sequences in the NCBI GenBank database using basic local alignment search tool (BLAST). The candidate strain was further identified by sequence analysis of *gyrB* gene sequence (approximately 1200 bp) [37]. 

### 5.5. Bacillus-Related Enterotoxin Detection

Nonhemolytic enterotoxin A (Nhe A), and nonhemolytic enterotoxin B (Nhe B) were detected by the *Bacillus* Diarrhoeal Enterotoxin Visual Immunoassay (BDE VIA™) (TECRA International Pty Ltd, Chatswood, Australia). Nhe B and hemolysin BL (HBL) were detected by the Duopath® Cereus Enterotoxins kit (EMD Millipore, Merck KGaA, Darmstadt, Germany). Cereulide was detected by the Singlepath® Emetic Tox. Mrk. Kit (EMD Millipore, Merck KGaA, Darmstadt, Germany).

### 5.6. ZEN Detoxification Capability Test in Culturing Condition (TSB Medium)

The ZDB strains were inoculated at 1% (*v/v*) in TSB containing 5 mg L^−1^ ZEN at 37 °C for 24 h. After that, samples were centrifuged (8000× *g* for 20 min at 4 °C) and supernatants were collected for the residual ZEN analysis. The ZEN detoxification of the ZDB strains was compared to a strain of *B. subtilis* (BCRC 17,441) from the Bioresource Collection and Research Center, Food Industry Research and Development Institute (Taiwan) to assess their relative detoxification capabilities [27].

### 5.7. ZEN Adsorption Ability and ZEN Degradation Ability

The ZDB strains were inoculated at 1% (*v/v*) in TSB at 37 °C for 24 h. Following the incubation, the cells and supernatants were separated by centrifugation (8000× *g* for 20 min at 4 °C). The cells were used for the ZEN adsorption ability test, and the supernatants were used for the ZEN degradation test. The separated cells were resuspended in 10 mL PBS containing ZEN (5 mg·L^−1^) and incubated with constant shaking at 150 rpm. After 30 min, the culture was centrifuged (8000× *g* for 20 min at 4 °C), and the supernatants were collected and analyzed for residual ZEN. The ZEN degradation test was carried out by adding ZEN to the collected supernatants (at a final concentration of 5 mg L^−1^) and incubated in a rotary shaking incubator (150 rpm) at 37 °C. After 24 h, the supernatant was analyzed for residual ZEN. 

### 5.8. Enzymatic Profile of ZDB Strains

The API ZYM system (bioMérieux, Marcy l’Etoile, France) was used for the assay of enzymatic activities of the ZDB strains. After subcultured twice, the cells of each ZDB strain were collected through centrifugation (8000× *g* for 20 min) and resuspended in API suspension medium with turbidity adjusted to 5–6 McFarland. All detection tests were performed according to the manufacturer’s instructions.

### 5.9. Detoxification of ZEN-Contaminated Maize by ZDB Strains

ZEN (5 mg·kg^−1^) was added to 20 g of sterile ZEN-free maize, and then 60 mL of sterile distilled water was added. The inoculum of the candidate strain was added at 1% (*v/v*) and incubated at 37 °C for 48 h. After 24 and 48 h, samples were collected for monitoring bacterial number and ZEN detoxification activity. Bacterial counts were performed with TSA plating at 37 °C for 24 h. Finally, all fermentation residues were collected, freeze-dried, and analyzed for residual ZEN according to Ok et al. [38].

### 5.10. The Effect of ZEN on the Candidate Strain (B2 Strain) Growth in TSB Medium

The candidate strain (B2 strain) was inoculated at 1% (*v/v*) in TSB with or without ZEN (at a final concentration of 5 mg·L^−1^), and incubated at 37 °C for 24 h. After 8 h and 24 h, samples were collected for checking bacterial numbers. At the end of incubation, the supernatants were collected and analyzed for the pH, NH_3_-N, lactic acid, acetic acid, and total volatile fatty acids (VFAs). Bacterial count was done as previously. The pH value was measured with a pH meter (pH 22, Horiba, Kyoto, Japan). The NH_3_-N concentration was determined as described by Chaney and Marbach [39]. The lactic acid concentration was determined by L-lactic acid assay kit (LC2653, Randox, Crumlin, UK). Acetic acid and total VFAs were analyzed using gas chromatography (GC7820A, Agilent, Santa Clara, CA, USA) with a flame ionization detector and the Nukol™ capillary GC column (size × I.D. 30 m × 0.25 mm, df 0.25 μm) (SUPELCO, Bellefonte, PA, USA). The carry gas was helium gas. The crotonic acid (25 g·L^−1^) was used as an internal standard.

### 5.11. The Effect of ZEN on Fermentation Characteristics of the Candidate Strain (B2) in Maize

The candidate strain (B2 strain) was inoculated at 1% (*v/v*) in ZEN-free maize or ZEN-contaminated maize (at a final concentration of 5 mg·kg^−1^) added 60 mL of sterile distilled water. The fermentation process was under aerobic conditions at 37°C for 72 h. After 48 h and 72 h, samples were collected for an analysis of bacterial count and pH value. After then, the supernatants were collected for analysis of NH_3_-N, lactic acid, and acetic acid. 

### 5.12. Other Mycotoxin (AFB1, DON, FB1 and T2 Toxin) Detoxification Test

The candidate strain (B2 strain) was inoculated at 1% (*v/v*) in individual TSB containing AFB1 (5 µg·L^−1^), DON (400 µg·L^−1^), FB1 (500 µg·L^−1^), or T2 toxin (100 µg·L^−1^), respectively. After incubated at 37 °C for 24 h, samples were centrifuged (8000× *g* for 20 min at 4 °C) and supernatants were collected for the residual mycotoxins analysis. Residual AFB1, DON, FB1, and T2 toxin were performed by using enzyme-linked immunosorbent assay kit (Vaccigen, New Taipei, Taiwan). 

### 5.13. Statistical Analysis

The data of the effect of ZEN on the candidate strain (B2 strain) growth in TSB, fermentation characteristics in maize experiments, and mycotoxin (AFB1, DON, FB1, and T2 toxin) detoxification capability test were analyzed via *t*-test analysis. The data of ZDB strains’ ZEN detoxification capability, adsorption ability, degradation ability, and detoxification of ZEN-contaminated maize experiment were analyzed by the general linear model procedure of SAS, Version 9.4 and expressed as the means ± standard deviation (SD) (SAS Institute Inc., Cary, NC, USA). Statistical differences were determined by Duncan’s multiple range test, and significance was defined as *p* < 0.05.

## Figures and Tables

**Table 1:** The characteristics of 14 isolates isolated from different sources with ZEN detoxification ability.

Strains	Source	Enterotoxins ^b^			Emetic Toxin	Code ^c^	16S rDNA Sequencing (Identify%)
		Nhe A	Nhe B	Hbl L2	Cereulide		
Isolate 1	Fermented soybean product	−	−	−	−	B1	*B. subtilis* (99)
Isolate 2	Fermented soybean product	+	−	−	−		
Isolate 3	Fermented soybean product	−	−	−	−	B2	*B. subtilis* (99)
Isolate 4	Fermented soybean product	+	−	−	−		
Isolate 5	Fermented soybean product	−	−	−	−	B3	*B. subtilis* (99)
Isolate 6	Fermented soybean product	−	−	−	−	B4	*Bacillus* sp. (100)
Isolate 7	Fermented soybean product	+	−	−	−		
Isolate 8	Sewage	+	−	−	−		
Isolate 9	Soil	+	−	−	−		
Isolate 10	Soil	+	−	−	−		
Isolate 11	Soil	+	−	−	−		
Isolate 12	Soil	+	+	−	−		
Isolate 13	Soil	−	−	−	−	B5	*Bacillus* sp. (100)
Isolate 14	Soil	+	−	−	−		

^a^ Enterotoxins included nonhemolytic enterotoxin A (Nhe A), nonhemolytic enterotoxin B (Nhe B), and hemolysin BL (HBL). The “−” and “+” represent negative and positive response, respectively. ^b^ Five nontoxic ZEN-detoxifying *Bacillus* (ZDB) strains were selected for further study.

**Table 2:** The ZEN detoxification capability, absorption, and degradation ability of ZEN-detoxification *Bacillus* strains in TSB medium.

*Bacillus* Strains	Detoxification Rate (%) *	Absorption Rate (%) *	Degradation Rate (%) *
BCRC 17,441 ^#^	51.9 ± 7.55 ^a^	47.0 ± 9.26 ^a^	31.0 ± 0.45
B1	41.8 ± 6.35 ^b^	29.0 ± 3.43 ^b^	24.0 ± 8.09
B2	41.4 ± 2.76 ^b^	26.5 ± 6.11 ^b^	35.0 ± 4.22
B3	28.1 ± 3.50 ^c^	29.6 ± 3.79 ^b^	27.2 ± 0.24
B4	58.1 ± 3.02 ^a^	24.7 ± 9.31 ^b^	31.0 ± 4.15
B5	43.1 ± 0.94 ^b^	30.4 ± 2.86 ^b^	32.8 ± 5.57

* The ZEN-detoxification *Bacillus* strains were inoculated in TSB containing 5 mg·L^−1^ ZEN and incubated at 37 °C for 24 h. The values represent the mean ± SD of triplicate experiments. Following the 24 h incubation, the cells and supernatants were separated by centrifugation. The separated cells were used for the ZEN adsorption ability test, and the collected supernatants were used for the ZEN degradation ability test. The values represent mean ± SD of triplicates. ^#^ *B. subtilis* (type strain BCRC 17,441) was used as standard strain for five ZDB strains to compare with. ^a,b,c^ Means in the same column with different superscript significantly differ (*p* < 0.05).

**Table 3:** Enzyme activities of ZEN-detoxification *Bacillus* strains checked by the API ZYM system.

Enzyme	BCRC 17,441	B1	B2	B3	B4	B5
Alkaline phosphatase	+ ^a^	+	+	++	+	++
Acid phosphatase	+	++	++	+	+	+
Esterase (C4)	+	++	++	+	+	+
Esterase lipase (C8)	+	+	+	++	+	+
Lipase (C14)	−	−	−	−	−	−
Leucine arylamidase	+	−	−	+	+	++
Valine arylamidase	+	−	−	+	+	+
Cystine arylamidase	−	−	−	+	−	+
Trypsin	−	−	−	−	−	−
α-chymotrypsin	+	−	−	+	+	+
Naphthol-AS-BI-phosphohydrolase	+	+	+	+	+	+
α-galactosidase	−	−	−	+	−	−
ß-galactosidase	+	+	−	+	+	+
ß-glucuronidase	−	−	−	−	−	−
α-glucosidase	+	−	−	++	+	+
ß-glucosidase	−	−	−	+	−	+
N-acetyl-ß-glucosaminidase	−	−	−	−	−	−
α-mannosidase	−	−	−	−	−	−
α-fucosidase	+	−	−	−	−	−

^a^ The “−” and “+” represent negative and positive response, respectively. The “++” represents stronger enzyme activity response.

**Table 4:** The bacterial count and detoxification rate of ZEN-detoxifying *Bacillus* strains in ZEN-contaminated (5 mg·kg^−1^) maize after 24 h and 48 h fermentation.

*Bacillus* Strains	Bacterial Count (log CFU mL^−1^)		Detoxification of ZEN in Maize after 48 h (%)
	24 h	48 h	
BCRC 17,441	9.67 ± 0.561 ^a^	8.69 ± 0.499 ^a^	32.7 ± 10.42 ^b,c^
B1	6.32 ± 0.922 ^b^	7.09 ± 0.379 ^b^	49.0 ± 9.43 ^a,b^
B2	9.44 ± 0.175 ^a^	9.20 ± 0.153 ^a^	55.8 ± 6.20 ^a^
B3	9.11 ± 0.494 ^a^	8.81 ± 0.438 ^a^	31.4 ± 10.96 ^c^
B4	8.21 ± 1.861 ^a^	8.79 ± 0.358 ^a^	49.2 ± 8.46 ^a,b^
B5	9.32 ± 0.210 ^a^	9.19 ± 0.516 ^a^	38.3 ± 3.57 ^b,c^

The values represent mean ± SD of triplicate experiments. ^a,b,c^ Means in the same column with different superscript significantly differ (*p* < 0.05).

**Table 5:** The characteristics of B2 strain in culture medium (TBS) without or with ZEN (5 mg·L^−1^) after 24 h incubation.

Measurement	−ZEN	+ZEN
Bacterial count (log CFU mL^−1^) (8 h)	10.4 ± 0.60	11.8 ± 0.58 *
Bacterial count (log CFU mL^−1^) (24 h)	10.3 ± 0.05	11.3 ± 0.14 *
pH	4.77 ± 0.042	5.64 ± 0.056 *
NH_3_-N (mmol L^−1^)	11.33 ± 0.891	27.57 ± 1.819 *
Lactic acid (mmol L^−1)^	208.2 ± 8.02	284.8 ± 20.02 *
Acetic acid (mmol L^−1^)	5.22 ± 0.269	11.55 ± 1.030 *
Total VFAs (mml L^−1^)	5.88 ± 0.325	12.41 ± 1.042 *

The values represent mean ± SD of quadruplicate experiments. * Significant difference (*p* < 0.05).

**Table 6:** The fermented characteristics of B2 strain in ZEN-contaminated (5 mg·kg^−1^) maize after 48 h and 72 h fermentation.

Measurement	−ZEN	+ZEN
Bacterial count (log CFU·mL^−1^) (48 h)	8.65 ± 0.334	8.99 ± 0.289
Bacterial count (log CFU·mL^−1^) (72 h)	8.84 ± 0.186	8.50 ± 0.327
pH (48 h)	4.58 ± 0.022 *	4.46 ± 0.070
pH (72 h)	4.35 ± 0.068	4.41 ± 0.079
NH_3_-N (mmol·L^−1^) (48 h)	6.03 ± 0.372	6.10 ± 0.259
NH_3_-N (mmol·L^−1^) (72 h)	5.97 ± 0.230	6.65 ± 0.269 *
Lactic acid (mmol·L^−1^) (48 h)	67.8 ± 13.51	96.8 ± 8.88 *
Lactic acid (mmol·L^−1^) (72 h)	88.8 ± 12.26	111.9 ± 15.33
Acetic acid (mmol·L^−1^) (48 h)	21.3 ± 0.18	19.0 ± 1.02
Acetic acid (mmol·L^−1^) (72 h)	21.6 ± 0.82 *	19.4 ± 0.62

The values represent mean ± SD of quadruplicate experiments. * Significant difference (*p* < 0.05).
